# Immunogenicity of Hepatitis B Virus Vaccination in Relapsing–Remitting Multiple Sclerosis Patients Under Immunocompromising Treatment

**DOI:** 10.3390/ijms27062801

**Published:** 2026-03-19

**Authors:** Jerónimo Cruces-Párraga, Ana Muñoz-Jurado, Begoña M. Escribano, Francisco A. Martín-Hersog, Clara Triguero-Ortiz, Claudia Carmona-Medialdea, Isaac Túnez, Javier Caballero-Villarraso, Eduardo Agüera-Morales

**Affiliations:** 1 Maimónides Biomedical Research Institute of Córdoba (IMIBIC), 14004 Córdoba, Spain; jeroparraga@gmail.com (J.C.-P.); b22mujua@uco.es (A.M.-J.); am1esdub@uco.es (B.M.E.); fr19an94mh@gmail.com (F.A.M.-H.); clara.triguero@salud.madrid.org (C.T.-O.); claudiacm88@hotmail.es (C.C.-M.); itunez@uco.es (I.T.); 2 Neurology Department, Reina Sofía University Hospital, 14004 Córdoba, Spain; 3 Department of Biochemistry and Molecular Biology, University of Córdoba, 14071 Córdoba, Spain; 4 Department of Cell Biology, Physiology and Immunology, University of Córdoba, 14071 Córdoba, Spain; 5 Clinical Analyses Service, Reina Sofía University Hospital, 14004 Córdoba, Spain

**Keywords:** multiple sclerosis, hepatitis B vaccine, disease-modifying therapies, immunisation, lymphopenia

## Abstract

Multiple sclerosis (MS) is an autoimmune and demyelinating disease of the central nervous system (CNS). By acting on the immune system, disease-modifying therapies (DMTs) can control disease activity, but they indirectly increase susceptibility to infections, so different vaccines are necessary to prevent it. DMTs may potentially affect vaccine-induced seroconversion. We aim to analyse the response to the hepatitis B virus (HBV) vaccine (Engerix-B) in relapsing–remitting MS patients (RRMS) using these therapies because the scientific literature remains limited in this area. A retrospective observational study of RRMS patients vaccinated against HBV was conducted. Acquired immunity after vaccination was determined, and an analysis of immunogenicity was conducted based on the type of DMT (immunomodulators/immunosuppressants), vaccine doses, total lymphocyte count (TLC), age, and sex. 200 patients were included, with a mean age 47.79 years, and 140 (70%) were women. A lower vaccine response was observed in patients treated with immunosuppressive DMTs (51.8%, *p* < 0.001), particularly with fingolimod (32.4%, *p* < 0.001), and a higher response was seen with immunomodulators like teriflunomide and interferon-β1a (100%, *p* < 0.001). Using logistic regression, a model was obtained that included the number of vaccine cycles, lymphopenia and type of DMT associated with the response to the HBV vaccine. It is necessary to adapt HBV vaccination protocols for MS patients, considering the type of DMT used and baseline immune status.

## 1. Introduction

Multiple sclerosis (MS) is a chronic, inflammatory, autoimmune, demyelinating, and degenerative disease of the central nervous system (CNS) [1] that results in motor, sensory and/or cognitive deficits [2,3]. It affects approximately 2.8 million people worldwide, with a higher prevalence in women than in men [4]. MS predominantly develops in young adults aged between 20 and 50 years and is the first cause of non-traumatic neurological disability in this population [5].

MS is a complex and multifactorial disorder in which clinical manifestations are highly heterogeneous in the severity, course and duration of symptoms [1,6]. Its clinical course is traditionally subdivided into three subtypes and the most common (85%) is relapsing–remitting MS (RRMS) [7], which is characterised by alternating periods of neurological dysfunction, known as relapses, and intervals of relative clinical stability, known as remissions [8]. Other subtypes are secondary progressive MS (SPMS) and primary progressive MS (PPMS) [8,9].

Despite the constant rising prevalence of this disease, there is still no cure. On one hand, acute treatment at the time of a relapse—primarily with corticosteroids—aims to accelerate clinical recovery without altering the long-term prognosis. On the other hand, to reduce the risk of further relapses, minimise long-term disease impact, and prevent disability progression [5,10], so-called disease-modifying therapies (DMTs) have been developed [7,10]. Classically, DMTs, whether oral or injectable (subcutaneous, intramuscular or intravenous) are classified as immunosuppressive or immunomodulatory according to their mechanism of action, although all aim to control neuroinflammation and therefore disease activity.

This mechanism of action makes sense if we understand that MS is primarily driven by immune-mediated mechanisms [11]. Diverse subsets of immune cells have distinct roles in the pathogenesis of multiple sclerosis. For example, MS is characterised by T regulatory cells (Treg) dysfunction, enhanced pro-inflammatory Th1 and Th17 responses and autoreactive B cell over activity [12].

B cells and plasma cells are present within inflammatory infiltrates of the central nervous system and can produce pathogenic autoantibodies that contribute to demyelinating injury. In addition, an intrathecal humoral response—evidenced by oligoclonal bands in the cerebrospinal fluid—is a characteristic feature of the disease [10].

Immunosuppressive drugs are inhibitors of crucial components of the immune system [13], while immunomodulators can shift immune responses from a pro-inflammatory toward an anti-inflammatory status by producing reversible or compartment-selective effects and generally milder impacts on vaccine immunogenicity [14,15].

The distinction between these DMT subgroups is based on the specific drug mechanism of action, depleting vs. non-depleting total lymphocyte count (TLC), depth/duration of lymphocyte reduction, other vaccine-response data, and infectious-risk signals [16,17,18]. Nevertheless, immunosuppressive or immunomodulatory effects may be common in the same drug [15,17]. According to the latest review by the Cochrane Collaboration, based on the class to which they belong and their mechanisms of action, drugs for the treatment of multiple sclerosis can be classified as follows [15]:

- Immunomodulatory agents:
  - Interferon-β1a: Its mechanism of action in MS is incompletely understood. Recombinant forms of interferon-β are believed to directly increase expression and concentration of anti-inflammatory agents, while downregulating the expression of pro-inflammatory cytokines [15,17,19].
  - Glatiramer acetate: It has an immunomodulatory action (anti-inflammatory) by inducing tolerance or anergy of myelin-reactive lymphocytes, without relevant changes in lymphocyte counts [14].
  - Dimethyl fumarate: It acts primarily by triggering the activation of a nuclear factor (Nrf2) transcriptional pathway, so it promotes anti-inflammatory activity and can inhibit expression of pro-inflammatory cytokines and adhesion molecules [15,17]. It induces the depletion of memory T cells, reduces the count of activated T cells, and supports the expansion of naive T cells [19].
  - Teriflunomide: Although it may cause systemic immunosuppression, teriflunomide is an immunomodulatory [14,20] agent with anti-inflammatory properties whose exact mechanism of action in MS is not fully understood [15,21]. The drug is thought to reduce the number of activated lymphocytes, which would cause inflammation and damage myelin in the central nervous system [14].
- Within the immunosuppressive DMTs, the following agents are included:
  - Fingolimod: It is a sphingosine-1-phosphate-receptor (S1PR) modulating agent who blocks egress of lymphocytes (mainly CCR7 + CD4+ naive and central memory T cells) from the lymph nodes [14,18].
  - Cladribine: Cladribine’s mechanism of action is closely linked to a reduction in lymphocyte count. It is a purine antimetabolite that provides a reduction in circulating T (CD4+, CD8+) and B-lymphocytes with relative sparing of other immune cells [17].
  - Alemtuzumab: It is a monoclonal antibody against the CD52 antigen expressed on lymphocytes and monocytes. Its effects in MS are thought to be mediated by an extended T and B lymphocyte depletion and change in the composition of lymphocytes that accompanies lymphocyte reconstitution [14,15]. B cell recovery is usually completed within 6 months. CD3+ and CD4+ lymphocyte generally do not return to baseline by 12 months post-treatment [17].
  - Ocrelizumab: It is a monoclonal antibody against the CD20 antigen expressed on B-lymphocytes. As CD20 is expressed on premature and memory B cells, but not on lymphoid stem cells, pre-existing humoral immunity due to plasma cells is preserved during ocrelizumab therapy [15,17].
  - Natalizumab: It is a humanised recombinant IgG antibody that impairs leukocyte extravasation into the CNS and intestinal tract by blocking the alpha-4 subunit of the integrin molecules on leukocytes. By this mechanism of action as a selective immunosuppressor, lymphocytes are not able to cross the blood–brain barrier, and inflammation in the CNS compartment is reduced [17,18,22]. Unlike other known DMTs, natalizumab does not have relevant effects on the CD4+/CD8+ ratio in peripheral blood, but it leads to a reduction in this ratio in the CNS [17].

However, a crucial consideration is that by acting on the immune system, most DMTs increase susceptibility to infections, causing more severe primary infections, and reactivation of latent pathogens and/or exacerbation of asymptomatic chronic infections, potentially leading to an increased risk of worsening clinical condition and to complications in terms of hospitalisation and mortality [23,24]. Accordingly, some patients with MS should be screened for active or latent infections and have their immunisation schedules updated before initiating these therapies. Recommended vaccines include those against hepatitis B, herpes zoster, influenza, human papillomavirus, tetanus–diphtheria, pneumococcal and meningococcal disease, and COVID-19 [24,25,26].

This recommendation poses challenges, particularly for highly active patients, because vaccination may require either a prolonged delay in treatment initiation or selecting a specific DMT during the vaccination period. For this reason, decisions about the optimal timing for vaccination should consider the patient’s clinical situation, the type of vaccine and DMT and the risk for suboptimal response to vaccination [26].

Moreover, it is well-known that vaccine effectiveness may be affected by DMTs because to treat the autoimmune mechanism of MS, these treatments can reduce both humoral and cellular immunity, leading to variability in immune responses, and indirectly, potentially affecting vaccine-induced seroconversion [19,27]. Indeed, evidence has shown the interactions between these therapies and seroprotection rates after influenza, pneumococcal [27] and SARS-CoV-2 vaccination [28,29]. However, the scientific literature on the effect of DMTs on the hepatitis B vaccine (HBV) remains limited [30,31].

This fact is particularly important because some of the RRMS patients included in our study had previously been vaccinated against hepatitis B, usually during childhood, in accordance with the recommendations of the current vaccination schedule in our country. However, baseline serology prior to inclusion in this study confirmed the absence of detectable serological immunity, defined by anti-HBs titres (antibodies directed against the hepatitis B surface antigen), below the seroprotection threshold (anti-HBs < 10 mIU/mL). Consequently, they are candidates for a revaccination schedule (described in Section 4), whose response could be influenced by the concomitant use of DMTs.

Therefore, the main objective of this study was to determine which factors influenced the seroprotection rate against HBV in individuals with RRMS, focusing on the influence of the type of DMT used during the vaccination cycles.

## 2. Results

A total of 200 RRMS patients were evaluated (Table 1); 140 (70%) were women. The population’s mean age was 47 years and ranged between 19 and 71 years.

Regarding the hepatitis B doses administered, 80.5% of the sample required a single vaccination cycle (*n* = 161) and the remaining 19.5% required two vaccination cycles (*n* = 39).

### 2.1. Therapeutic Group and Seroprotection

Table 2 shows the three therapeutic groups that are of interest to us. The percentage of seroprotected patients was calculated for each of these subgroups and also for the specific drugs that comprise them.

We observed that in the group of naive patients (*n* = 26), the seroprotection rate was 80.8% (*n* = 21); among the 85 patients undergoing treatment with immunosuppressive DMT, the proportion was 51.8% (*n* = 44) and the highest seroprotection rate was found in the group of people undergoing treatment with an immunomodulatory drug, at 94.4% (*n* = 84).

**Figure 1:**
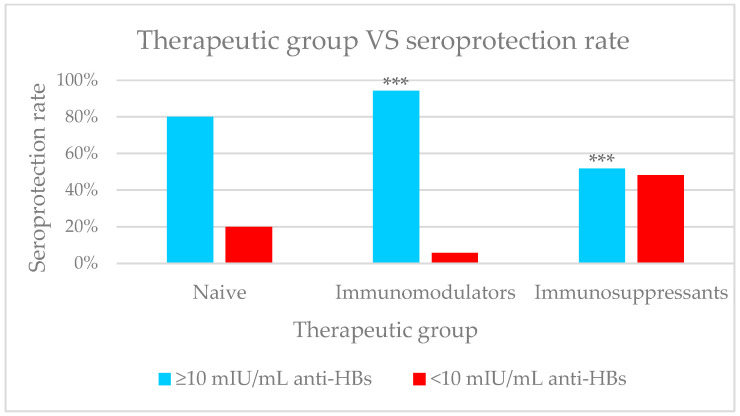
Overall sample population. Comparison between therapeutics groups and seroprotection rate. The global comparison among treatment groups was significant (χ^2^, *p* < 0.001). Pairwise post hoc analyses revealed higher seroprotection in patients receiving immunomodulators compared with immunosuppressants (*p* < 0.001). Statistical significance denoted by *** *p* < 0.001.

Figure 1 shows that the proportion of seroprotected patients presented statistically significant differences between the three groups (80.8% vs. 51.8% vs. 94.4%; (χ^2^, *p* < 0.001). Additionally, there were significant differences between patients treated with immunosuppressive and immunomodulatory DMTs (51.8% vs. 94.4%; χ^2^, *p* < 0.001).

Based on standard clinical practice in the Neurology Department of our hospital (Reina Sofía University Hospital, Cordoba, Spain), the most used drugs were as follows:

Among immunomodulatory drugs, DMF (n = 42; 47.4%) was the most widely used DMT. All drugs belonging to this class had a seroprotection rate of over 90%.

Among immunosuppressants, fingolimod (n = 34; 47.4%) and natalizumab (n = 33; 47.4%) were the two most used drugs.

Additionally, given that we observed that natalizumab had a higher seroprotection rate among immunosuppressive drugs, a sub-analysis of this response rate was performed for the following groups:

- Immunosuppressants, including natalizumab: 44/85 (51.8%)
- Immunosuppressants, excluding natalizumab: 18/52 (34.6%)
- Natalizumab: 26/33 (78.8%).

**Figure 2:**
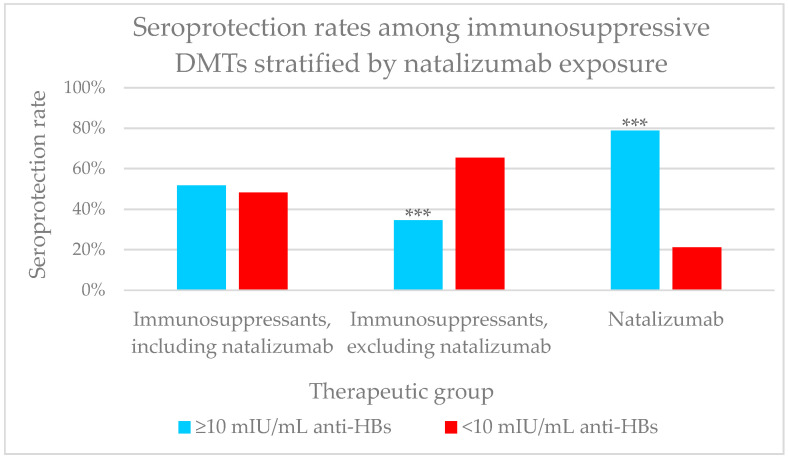
Seroprotection rates among immunosuppressive DMTs stratified by natalizumab exposure. We observed a higher seroprotection rate in patients receiving natalizumab compared to patients treated with the other immunosuppressants (χ^2^, *p* < 0.001). Statistical significance denoted by *** *p* < 0.001.

Due to its selective mechanism of action, when comparing seroprotection rates between natalizumab and other immunosuppressive drugs (alemtuzumab, fingolimod, cladribine, and ocrelizumab), we observed significant differences between the two subgroups (78.8% vs. 34.6%, *p* < 0.001) (Figure 2).

### 2.2. Other Factors That Could Influence Seroprotection

#### 2.2.1. Sex and Age

In the overall sample population (*n* = 200), irrespective of DMT exposure, women did not exhibit a higher vaccine response than men (77.9% vs. 66.7%; *p* = 0.096). Among patients receiving a DMT during vaccination (n = 174), seroprotection did not differ by sex (women 76.9% vs. men 66%; *p* = 0.136).

As explained in Section 4, a cut-off point was established at 55 years old, and based on this, patients were subdivided into two groups (above and below that age).

A total of 154 individuals were aged 55 or younger, and in this subgroup, 72.1% (*n* = 111) were women and 27.9% (*n* = 43) were men. On the other hand, 46 patients were over 55 years of age, of whom 63% were women (*n* = 29) and 37% were men (*n* = 17). We observed no differences in seroprotection between the two age groups (76.6% vs. 67.4%, *p* = 0.207).

#### 2.2.2. Number of Vaccine Doses and Vaccine Cycles

The number of patients requiring one or two vaccination cycles, the total number of doses administered within each cycle, and the corresponding stratified seroprotection proportion for each subgroup are summarised in Table 3.

A total of 162 people had received a single vaccination cycle. Of these, 100 (61.7%) patients had received three doses of the vaccine, and 62 (38.3%) people had received four doses.

On the other hand, 38 people had received two vaccination cycles. This means that they required extra doses of the vaccine and, as a result, 22 (57.9%) people needed six doses, and 16 (42.1%) people received seven doses.

Firstly, if we compare the percentage of seroprotection according to the number of cycles, we found that there were significant differences between the two groups. The percentage of patients with protective serology was 84% when they received only one cycle, compared to 34.2% when they needed two series of vaccinations, with statistically significant differences between the two groups (*p* < 0.001).

Additionally, regarding patients who had received a single cycle, there were no differences between administering three or four doses (81% vs. 88.7%, *p* = 0.194). In the subgroup with two vaccination cycles, there were also no differences between administering six or seven doses (36.4% vs. 31.3%, *p* = 0.743).

In Section 2.2.1, we report that sex and age do not condition significant differences in the overall seroprotection rates of the patients studied. Nevertheless, to better understand why 65.8% of these 38 patients who underwent a second HBV vaccination course did not respond, we reanalysed the effects of age, sex, and DMT exposure within this subgroup. Once again, using Fisher’s exact test, no differences were observed in the proportion achieving seroprotection when stratified by age or sex (*p* = 0.118 and *p* = 0.744, respectively).

However, when we analysed the type of DMT used, we found that 34 (89.5%) of the 38 people who underwent a second vaccination cycle were still being treated with immunosuppressive drugs and that seroconversion was particularly low (26.5%).

**Figure 3:**
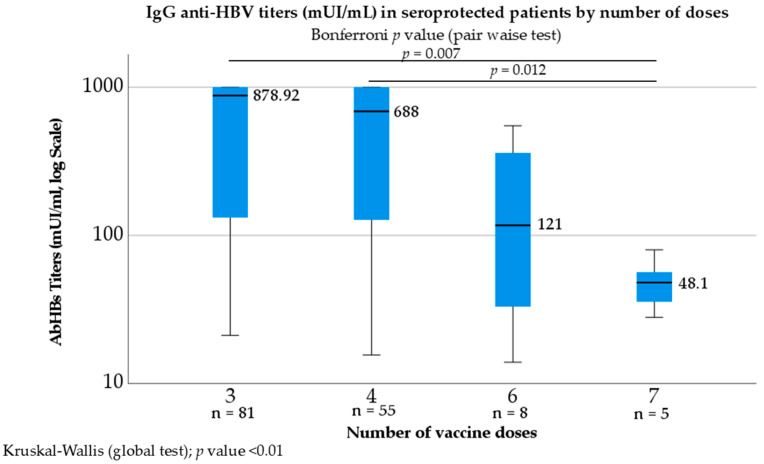
Box plot showing IgG anti-HBV titres (mUI/mL) in seroprotected patients according to the number of doses. Boxes represent the interquartile range (IQR), horizontal lines, the median values, and whiskers the minimum and maximum values. Median titres are indicated above each box. Differences across groups were evaluated using the Kruskal–Wallis test (global *p* < 0.01), with a significant pairwise difference in Bonferroni-adjusted post hoc tests.

In a secondary analysis (Figure 3), anti-HBs titres were compared by total doses received. Among the 149 patients with seroprotection (IgG anti-HBs ≥ 10 mIU/mL), the median (IQR) IgG anti-HBs titres was 567.45 mIU/mL (87.5–1000.00). There were four subgroups in which three doses (*n* = 81), four doses (*n* = 55), six doses (*n* = 8) and seven doses (*n* = 5) were administered. Across the four dose groups, titres differed significantly (Kruskal–Wallis H (3) = 16.739; *p* = 0.004; ε^2^ = 0.071). In Bonferroni-adjusted post hoc tests, titres were significantly lower in the seven-dose group than in the three- and four-dose groups (*p* = 0.007 and *p* = 0.012, respectively).

An ordered-trend test (Jonckheere–Terpstra) confirmed a downward gradient (Z = −2.509; *p* = 0.012, two-sided; *r* = 0.206), consistent with lower titres at higher positions in the ordering. Median (IQR) titres were 878.92 (131.50–1000) for three doses, 688.00 (115–1000.00) for four doses, 121.00 (29.54–398.58) for six doses, and 48.10 (31.85–68.29) for seven doses.

When stratified by number of doses, we observed that individuals who required three or four doses had antibody levels between 100 and 1000 mIU/mL, which was a normal response to the vaccine. However, patients who received six or seven doses of the vaccine had antibody titres in the range of 10–100 mIU/mL, which implied a low response to the vaccine.

#### 2.2.3. Total Lymphocyte Count

Among the 39 subjects (19.5%) with TLC ≤ 800, seroconversion was achieved in 28.2%. In contrast, among the 161 patients with TLC > 800, the seroconversion rate reached 85.7%, with a significant difference between subgroups (*p* < 0.001) (Table 4).

Most patients with lymphopenia (*n* = 34, 87.2%) were receiving immunosuppressive DMTs. Additionally, compared with patients whose TLC > 800, those with lymphopenia were more likely to require two vaccination courses (*n* = 19/161, 11.8% vs. *n* = 19/39, 48.7%; *p* < 0.001) and had lower seroprotection rates. Previously, we demonstrated that patients needing > 4 doses (i.e., two cycles) had reduced responses; in this sub-analysis, the proportion achieving seroprotection was even lower when lymphopenia was present.

### 2.3. Logistic Regression

We included all the variables previously analysed individually to try to predict the probability of seroconversion.

In order to identify potential confusing factors, we verified that removing sex and age from the estimated logistic regression models did not alter disrupt the coefficients of the remaining variables compared with the model that included them.

Finally, we obtained the following model with its corresponding equation:

$$P\;(\mathrm{seroprotection})\;=\;\;\frac{1}{1+\;e^{-(-1.286-1.457\times cycle+2.102\times TLC\;range-0.027\times DMTCAT1+\;1.734\times DMTCAT2)}}$$

A chi-square omnibus test was significant, rejecting the null hypothesis that all β coefficients (except the intercept) were equal to zero. The coefficient of determination (R^2^) indicated that 47.6% of the variation in the dependent variable was explained by the predictors. In the final binary logistic regression, we included all the variables previously analysed individually to try to predict the probability of seroconversion with four variables, and the overall correct classification was 85.5%. The area under the ROC curve (AUC) was 0.854, which indicated a good discriminative ability.

Moreover, to quantify the contribution of each predictor to the logistic regression model, we conducted likelihood-ratio tests using nested models. Removing TLC (LR χ^2^(1) = 19.33, *p* < 0.001), Number of cycles (LR χ^2^(1) = 8.38, *p* = 0.004), or the DMT class variable as a whole (LR χ^2^(2) = 12.92, *p* = 0.002) significantly worsened the model fit.

**Figure 4:**
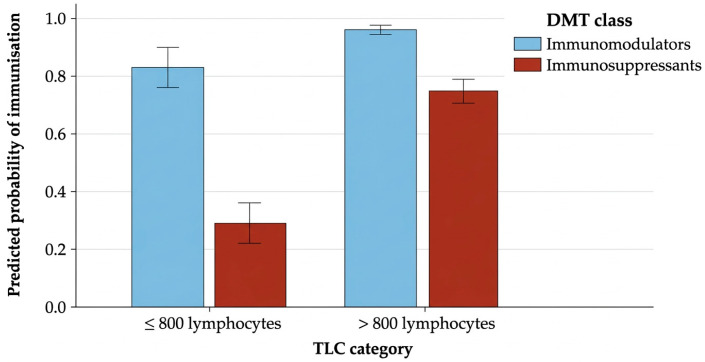
Predicted probabilities of achieving immunisation according to lymphocyte count category (<800 vs. ≥800 cells/µL) and DMT class (immunomodulators and immunosuppressants). Abbreviations: TLC, total lymphocyte count; DMT (disease-modifying therapies). Bars represent predicted probabilities derived from the logistic regression model with 95% confidence intervals. The naive group (*n* = 26) was excluded from the graphical representation due to its small sample size, which resulted in excessively wide confidence intervals and hindered visual interpretation.

This indicated that the probability of HBV seroprotection was related to the number of vaccination cycles, DMT class and the presence of lymphopenia (Table 5) (Figure 4).

## 3. Discussion

As previously noted, vaccination in patients with RRMS receiving DMTs is safe and necessary [23,24]. However, although several studies have demonstrated reduced responses to certain vaccines in this population due to concomitant DMT use, few have focused specifically on the hepatitis B vaccine [27,32]. Accordingly, we have evaluated the impact of different DMTs on the seroprotection rate achieved after administration of the hepatitis B vaccine, as well as the potential influence of sex, age, total lymphocyte count (TLC), and number of vaccine doses

### 3.1. Influence of Different DMTs

As explained in Section 4, patients were classified according to the treatment received in immunosuppressive DMTs or immunomodulatory DMTs.

The immunomodulatory subgroup includes DMTs that alter signalling, trafficking, or cytokine balance without targeted systemic depletion, producing reversible or compartment-selective effects and generally milder impacts on vaccine immunogenicity (e.g., interferon-β, glatiramer acetate, teriflunomide). By this reason, and as we show in our results, lower vaccine response has been demonstrated in patients receiving immunosuppressive DMTs compared with immunomodulatory DMTs [25,26,30,31,33]. In fact, recent consensus guidelines recommend initiating vaccination—even using accelerated schedules [34]—before starting immunosuppressive therapies [16,26].

Moreover, as reported in previous studies, comparisons have typically contrasted so-called injectable versus non-injectable agents [30,31,33]. Among the injectable agents, glatiramer acetate and interferon-β1a are notable; these agents scarcely affect TLC and immunogenicity to HBV vaccine.

Several authors report that patients treated with non-injectable DMTs like DMF exhibit lower seroprotection rates than those without DMT or those receiving injectable DMTs [17,30,35]. For example, vaccine responsiveness is maintained with moderately effective immunomodulatory therapies such as interferon-β1a [35] and teriflunomide [30,36].

Consistent with previous evidence, our findings in Table 2 support that vaccine response is preserved with teriflunomide or interferon-β1a and reduced with fingolimod.

Given its mechanism of action, fingolimod significantly decreases the absolute numbers of all major lymphocyte subsets, except for NK cells. The reduction is most pronounced within T helper and B cell populations, and it can reduce lymphocyte counts by 70–80% within 4–6 h after administration [17]. In our sample population, before initiation of the vaccination series, 34 subjects were receiving this agent. In this subgroup, 67.6% (*n* = 23) had no seroconversion; among these, 79.4% (*n* = 27) had a TLC < 800.

Therefore, among fingolimod-treated patients, the lack of seroconversion was frequent and most non-responders had lymphopenia (<800/µL). These findings support that under S1PR modulators, low circulating lymphocyte counts are associated with reduced humoral vaccine responses.

Our findings are consistent with a randomised trial [37], showing that, in fingolimod-treated non-responders after two COVID-19 vaccine doses, temporarily stopping fingolimod until TLC > 1000/µL before the third dose increased seropositivity and IgG levels.

Among immunosuppressed patients receiving infusion therapies such as ocrelizumab or alemtuzumab, our findings aligned with the available evidence, with loss of seroprotection observed in nearly half of cases [34,36]. Indeed, other authors recommend attempting to complete ≥3–4 HBV vaccine doses before initiating these drugs; when time is limited, an accelerated schedule may be considered [34].

By contrast, despite belonging to this therapeutic category and given its mechanism of action (selective immunosuppressant), natalizumab has not been associated with reduced vaccine immunogenicity in prior reports [17,22,35]. Consistent with this evidence, most natalizumab-treated patients in our cohort achieved seroprotection (78.8%), with statistically significant differences (*p* < 0.001) when compared to the group receiving other immunosuppressants.

In this regard, there is evidence showing that for people with highly active MS who require both immunisation and high-efficacy therapies, natalizumab treatment may be used as a bridging therapy while vaccination schedules have been completed, in order to prevent treatment delays [38]. Our research corroborates this role of natalizumab.

Finally, the greater vaccine responsiveness observed with interferon-β1a is consistent with a recent study in patients with MS receiving the SARS-CoV-2 vaccine. This study demonstrated for the first time that Interferon-β1a treatment enhanced the vaccine-specific humoral response compared to healthy controls, plausibly owing to the well-known preservation of B and T-lymphocytes with this agent [39]. Our results may suggest that this effect extends to HBV vaccination, and this hypothesis may be interesting for future studies.

### 3.2. Influence of Age and Sex

Age is a determinant factor of vaccine responsiveness [40], and its cut-offs are different across the literature, reflecting biological considerations (immunosenescence) and operational choices for subgrouping. Some studies compare groups using 50 years [30], whereas others use 55 years [31,33]. Ladeira et al. [33] argue that increasing age is associated with lower seroprotection, and Faustino et al. [30] report that among patients aged > 50 years who achieve seroprotection, post-immunisation IgG titres are lower, and a higher proportion requires a fourth dose of hepatitis B vaccine. Notably, that fourth dose would still form part of the primary vaccination series (cycle 1), and importantly, the final seroprotection status (positive vs. negative) did not differ between those aged >55 years and younger patients.

We compared outcomes by sex, as prior studies suggest that sex may influence responses to several vaccines (including HBV) through hormonal and epigenetic mechanisms [33]. In our sample population, however, we found no significant differences between women and men.

### 3.3. Influence of Total Lymphocyte Count

In general, developing protective immunity requires mounting an adaptive immune response to the vaccine through B- and T-lymphocytes. Specific antigens stimulate B- and T-lymphocytes, inducing clonal expansion and differentiation into effector populations and long-lived memory cells [41,42]. CD4+ T cells play a significant role in the production of antibodies during infection [41,43] and this process after hepatitis B vaccination is tightly regulated by both Th1 and Th2 [42].

It is well-known that high-efficacy DMTs are associated with lymphocyte depletion involving T cells, B cells, or both [37]. A significant decrease in the antibody level after vaccination is associated with the presence and degree of lymphopenia [37,43,44]. For all these reasons, we reviewed complete blood counts obtained before initiation of the vaccination series to determine each patient’s total lymphocyte count (TLC).

Our findings reinforce the role of total lymphocyte count as a key determinant of seroconversion capacity following HBV revaccination. Patients with TLC ≤ 800 had markedly lower seroprotection rates compared to those with TLC > 800, suggesting that lymphopenia significantly limits the ability to generate a protective serological response. Furthermore, the association between lymphopenia and the use of immunosuppressive DMTs, as well as the greater need to complete two vaccination cycles in this subgroup, indicate that we could anticipate a poor response to the vaccine in patients with these characteristics.

### 3.4. Number of Vaccine Doses

When we assessed seroprotection based on number of vaccine doses, we found no differences in seroconversion rates or IgG anti-HBs titres (mIU/mL) between patients receiving a three-dose series (0, 1, 6 months) and those receiving a four-dose series (0, 1, 2, 6 months), so our results are consistent with the findings of other studies [45]. Both schedules are valid and effective; the choice should be individualised based on whether the patient is already receiving a DMT and on timing considerations.

In our research, however, compared with patients who received two HBV vaccination courses (*n* = 38), those completing a single primary course (*n* = 161) showed a higher seroconversion rate (84.0% vs. 34.2%; *p* < 0.01).

It is well-known that there are various factors that can determine the capacity for immunisation following vaccination against hepatitis B. In fact, in a meta-analysis of 37 studies involving more than 21,000 adults, Yang et al. [40] observed that additional factors such as smoking or BMI were significantly associated with a lower seroconversion rate (anti-HBs < 10 mIU/mL). Moreover, various sources [46] indicate that the age of primary vaccination (and, therefore, the interval until adult revaccination) does influence the long-term persistence of anti-HBs, making it a factor to consider. For example, previous studies have shown that the rate of antibody loss is inversely correlated with the age of initial vaccination [47].

However, when analysing the response to the booster dose administered in adulthood (at age 30) in a multivariable model, the 30-year study found no association between age at primary vaccination and the probability of achieving a protective response to the booster [46]. Instead, those studies identified pre-booster anti-HB levels as the key determinant of booster response: participants with pre-booster anti-HB levels of 2–9.9 mIU/mL had a higher probability of response than those with levels < 2 mIU/mL [46]. This implies that, although the passage of time reduces titres, the most immediate factor determining whether immunological memory will be successfully reactivated is whether detectable antibodies remain, even if they are below the protective threshold.

Therefore, in searching for additional factors that could explain the low seroprotection achieved in our group of patients who underwent two vaccination cycles, we found that our seven-dose cohort exhibited both a lower probability of seroconversion and lower post-vaccination titres than the three-, four-, or six-dose cohorts. This does not imply that additional doses reduce immunogenicity; rather, it likely reflects that patients who required many doses were those with intrinsically poor vaccine responsiveness (e.g., due to host factors or concurrent therapies), and thus accumulating doses did not overcome that fact. From a clinical point of view, it is important as antibody titres before and after vaccination schedule seem to predict durability. Prior longitudinal studies show that higher initial IgG anti-HBs (especially >100 and >1000 mIU/mL) may be associated with longer persistence of protection over decades [47,48,49].

Therefore, patients who need multiple courses and still achieve only low titres are less likely to sustain immunity long-term and may benefit from alternative strategies such as higher-dose regimens or timing relative to immunosuppressants instead of simply more doses of the same vaccine.

There are studies comparing the immunogenicity obtained in the revaccination of patients, considering the use of Fendrix vs. Engerix-B, with improved seroconversion rates with the first vaccine [50]. We believe that these evaluations may contribute to improving the seroconversion rate in patients with greater resistance to the vaccine.

### 3.5. Limitations

It is important to acknowledge several limitations. First, although most patients were vaccinated with Engerix-B 20 μg, the specific vaccine administered was not known for all MS patients. Given that immunogenicity varies across HBV vaccines, this may have influenced our results and limited comparability with more homogeneous cohorts from other centres.

To enhance statistical power and internal homogeneity, we believe it would be valuable to expand the sample size for the subgroup of patients who receive six or seven vaccine doses and yet fail to achieve seroconversion. Although uncommon, these special cases could contribute to a better understanding of the factors underlying intrinsic vaccine non-responsiveness in some individuals.

## 4. Materials and Methods

### 4.1. Primary and Secondary Objectives

The main objective of this study was to determine which factors influenced the seroprotection rate following hepatitis B vaccination (Engerix-B) in individuals with RRMS, focusing on the influence of the type of DMT used: immunomodulators and immunosuppressants.

Secondarily, we aimed to analyse potential differences by age, sex, the specific type of drug used, vaccination cycles, and total lymphocyte count.

### 4.2. Study Design

The retrospective study included 200 consecutive patients who were being followed up at the Neurology Department of Reina Sofía University Hospital (Córdoba, Spain).

Patients were classified according to the treatment received before starting their vaccination programme into three categories (naive patients, immunomodulatory DMTs and immunosuppressive DMTs). All patients initially classified as naïve remained untreated, and all individuals who had previously received specific treatment continued with the same DMT throughout the vaccination process. Thus, the patients’ status never changed during follow-up.

We followed the classification of the latest Cochrane review [15] and specific references mentioned in Section 1.

- Naive patients: No prior DMT.
- Immunomodulatory DMTs: Interferon-β1a, glatiramer acetate, dimethyl fumarate and teriflunomide.
- Immunosuppressive DMTs: Fingolimod, cladribine, alemtuzumab, ocrelizumab and natalizumab.

We analysed the factors that influenced the seroprotection rate and, to this end, we evaluated the influence of the type of DMT, age, sex, vaccination cycles and total lymphocyte count in different sections.

In the specific case of Natalizumab, due to the implications that its specific mechanism of action as a selective immunosuppressant at the CNS level could have on vaccination [17], a sensitivity analysis was performed in the group of immunosuppressive drugs, with the aim of quantifying the seroprotection rate obtained in patients treated with Natalizumab, compared to the other DMTs in this group.

According to other previous studies [31,33] a cut-off point was established at 55 years old, and based on this, patients were subdivided into two groups (above and below that age). Moreover, before initiation of the vaccination series, TLC thresholds were defined according to prior reports [17,26] with lymphopenia set at ≤800.

### 4.3. Inclusion Criteria

- Age ≥ 18 years.
- RRMS diagnosis according to McDonald criteria [8].
- Naïve patients or people with any prior DMT before hepatitis B (HBV) vaccination.
- RRMS patients previously vaccinated against hepatitis B with absence of detectable serological immunity (anti-HBs < 10 mIU/mL). Consequently, they are candidates for a revaccination schedule.
- Complete HBV revaccination series: three-doses (0, 1, 6 months) or four-doses (0, 1, 2, 6 months).
- Blood test of pre- and post-vaccination serology available.

### 4.4. Exclusion Criteria

Patients were excluded if they had other MS subtypes or pre-existing hepatitis B immunity (either from prior vaccination or past infection) based on pre-vaccination serology.

Patients who changed DMT during the vaccination schedule or those initially naïve patients who started treatment during this process were also excluded.

### 4.5. Vaccination Programme

Pre-vaccination serology studies were performed for the following markers: hepatitis B virus surface antigen (HBsAg), anti-hepatitis B virus core (anti-HBc) antibodies and anti-hepatitis B virus surface antigen (anti-HBs) antibodies. HBV immunisation was recommended to all seronegative patients.

Recombinant DNA vaccines such as Engerix-B or Recombivax HBV, first developed and marketed in the 1980s, are administered following administration guidelines of three doses (0–1–6 months) or four doses (0–1–2–6 months) [51].

However, in the 2000s, enhanced immunity vaccines were developed, which may be administered to patients who were already immunosuppressed or who were going to start such treatment in the next 6 months, to optimise the vaccine response. Among these, we can highlight the Fendrix vaccine, which may be administered at 0–1–2–6 months [16].

Since our hospital belongs to the public health system, vaccines are purchased in batches of the same brand. For this reason, the only vaccine available for patients included in our study was Engerix-B.

Participants received a standard schedule with the Engerix-B 20 μg vaccine and the complete primary vaccination was defined as the number of doses recommended at the time of the patient’s initial assessment, comprising either three doses of 20 μg (administered at months 0, 1 and 6) or four double dose of the HBV vaccine containing 40 µg of antigen (administered at months 0, 1, 2 and 6). Both the single- and double-dose HBV vaccines were adequately immunogenic, and the double-dose HBV vaccine was not significantly more immunogenic than the single-dose vaccine in terms of the seroconversion rates [52].

Anti-HBs titres were reevaluated 4 weeks after the last dose. Titres ≥ 10 mIU/mL were considered positive. Accordingly, patients were classified as responders if IgG anti-HBs titres were ≥10 mIU/mL and non-responders if <10 mIU/mL [46,49].

In case of insufficient response, a complete revaccination was recommended [16], so non-responder patients underwent revaccination (second cycle) with three or four more doses (six, seven or eight doses total when accounting for the original dose series) [39,51].

Moreover, response categories were defined according to anti-HBs titres upon completion of the HBV vaccination series [47,49]:

- Low response: 10 mIU/mL ≤ anti-HBs < 100 mIU/mL.
- Normal response: 100 mIU/mL ≤ anti-HBs <1000 mIU/mL.
- A high response: anti-HBs ≥ 1000 mIU/mL.

To account for a ceiling effect at 1000 mIU/mL, values reported as ‘≥1000 mIU/mL’ were top-coded to 1000 mIU/mL.

### 4.6. Statistical Analysis

Descriptive analyses were presented as mean (±standard error of the mean), median and interquartile range (IQR). Categorial variables were described as frequency and percentage. For bivariate analyses, appropriate parametric or non-parametric tests were applied according to the data were normally distributed, as assessed by the Kolmogorov–Smirnov test. Chi-square test was used for qualitative variables (Pearson’s chi-square when sample size assumptions were met, and Fisher’s exact test when expected cell counts were small). Student’s *t*-test or Mann–Whitney U tests were applied for quantitative variables. Any two-sided *p* value lower than 0.05 was considered significant.

Finally, a binary logistic regression model was used to examine the association between achieving HBV seroprotection and potential determinants.

Independent variables included in all models were sex, age, number of vaccination cycles, total lymphocyte count range (TLC ≤ 800 vs. >800) and DMT class (immunomodulators vs. immunosuppressants). Given that a primary objective of our study was to compare seroprotection rates across DMT classes, the original three-level DMT class variable (naive, immunomodulator, immunosuppressive) was entered into the multivariable logistic model using indicator (dummy) coding (k − 1 variables). Specifically, DMT_cat1 identified naive patients and DMT_cat2 identified patients receiving an immunomodulatory therapy, with the immunosuppressor group serving as the reference category to enable a direct immunomodulator-versus-immunosuppressor comparison.

All possible interactions and potential confounders were considered. Goodness-of-fit was examined using the likelihood-ratio test and Nagelkerke’s R^2^. Discrimination was assessed by the area under the Receiver Operating Characteristic (ROC).

To quantify the incremental contribution of each predictor to the multivariable logistic regression model, we used likelihood-ratio (LR) testing based on nested models. Specifically, we compared the full model to reduced models in which one covariate was removed at a time and calculated the likelihood-ratio statistic as the difference in −2 log-likelihood (Δ−2LL). The resulting statistic followed an approximate chi-square distribution with degrees of freedom equal to the number of parameters removed.

Statistical analysis was performed using the software SPSS version 28.0 and Microsoft Excel v.18.24.

### 4.7. Ethical Concerns

Authorization was obtained from the Biomedical Research Ethics Committee of the province of Córdoba on 29 March 2022 (Code VHEBEM21; Reference Number 5229). Informed consent was obtained from each patient. The harmonised tripartite standards of the Helsinki declaration, the Organic Law on Biomedical Research of 15/2007 of 3 July, the Organic Law on Personal Data Protection (LOPD) of 13 December 2018, the code of ethics of the “Organización Médica Colegial” (OMC), the basic regulatory law 41/2002 on patient autonomy and rights and obligations regarding clinical information and documentation, of November 14, as well as the standards of good clinical practice were respected.

## 5. Conclusions

The probability of HBV seroprotection is related to the number of vaccination cycles (one vs. two cycles), DMT class (immunomodulators vs. immunosuppressants) and the presence of lymphopenia.

Natalizumab is a selective immunosuppressant that has a minor effect on the seroprotection rate achieved after vaccination against hepatitis B, so it can be used as bridging therapy in patients with highly active disease while vaccination is completed.

Moreover, the lower seroprotection rates achieved in patients treated with immunosuppressive drugs reinforce the importance of attempting to vaccinate these individuals before starting such treatments. Additionally, we know that there is a subgroup of people who, depending on the type of DMT used and the existence of lymphopenia, may have greater difficulty responding to the vaccine. We have found that, in these patients, after six to seven doses, seroprotection rates are low and antibody titres are lower.

Therefore, it makes sense to limit the number of vaccine doses administered to a maximum, beyond which we know it would be very difficult to achieve seroprotection. This would mean savings in economic terms and less discomfort for the patient, who could avoid receiving vaccine doses that would not improve the results.

## Figures and Tables

**Table 1:** Study population characteristics.

Characteristic	N = 200
*Age, mean (SD) (years)*	47.79 (9.37)
*≤55 years*	154
*>55 years*	46
*Sex, n (%)*	
Female	140 (70%)
Male	60 (30%)
*EDSS median (IQR)*	2.5 (3.5)
*Therapeutic group, n (%)*	
Naïve patients	26 (13%)
Immunomodulatory DMT	89 (44.5%)
Immunosuppressive DMT	85 (42.5%)
*Vaccine cycles, n (%)*	
1	161 (80.5%)
2	39 (19.5%)
*Vaccine doses, n (%)*	
3	100 (52.5%)
4	61 (28.5%)
*6*	23 (11.5%)
*7*	16 (8%)
*Seroprotection, n (%)*	
Positive	149 (74.5%)
Negative	51 (25.5%)

**Table 2:** Therapeutic group, specific DMT and seroprotection rate.

Therapeutic Group	Specific DMT	Seroprotection Rate for Each Specific DMT	Seroprotection Rate for Each Therapeutic Group
Naive *n* = 26	-	-	*n* = 21 80%
Immunomodulators *n* = 89	Teriflunomide (*n* = 19)	18 (94.7%)	*n* = 84 94.4% ***
	Interferon-β1a (*n* = 19)	19 (100%)	
	Dimethyl fumarate (DMF) (*n* = 42)	38 (90.5%)	
	Glatiramer acetate (*n* = 9)	9 (100%)	
Immunosuppressants *n* = 85	Alemtuzumab (*n* = 9)	4 (44.4%)	*n* = 44 51.8% ***
	Cladribine (*n* = 5)	3 (60%)	
	Fingolimod (*n* = 34)	11 (32.4%)	
	Natalizumab (*n* = 33)	26 (78.8%)	
	Ocrelizumab (*n* = 4)	0 (0%)	

Statistical significance denoted by *** *p* < 0.001.

**Table 3:** Seroprotection by number of HBV vaccination cycles and doses.

Number of Cycles	Number of Doses	Seroprotected by Number of Doses	Seroprotected by Number of Cycles
One cycle *n* = 162	3 (100)	81 (81%)	135 (84%)
	4 (62)	55 (88.7%)	
Two cycles *n* = 38	6 (22)	8 (36.4%)	14 (34.2%)
	7 (16)	5 (31.3%)	

**Table 4:** Total lymphocyte count and seroprotection rate.

TLC Range	*n*	Seroprotection by TLC
≤800	39 (19.5%)	11 (28.2%) ***
>800	161 (80.5%)	138 (85.7%) ***

Statistical significance denoted by *** *p* < 0.001.

**Table 5:** Logistic regression model.

Variable	Coefficient (β)	Standard Error	*p*-Value
Number of cycles	−1.457	0.509	0.004
Total lymphocyte count (TLC) range	2.102	0.496	<0.001
DMT_cat1 (naive)	−0.027	0.626	0.966
DMT_cat2 (modulator)	1.734	0.565	0.002

Omnibus χ^2^: 68.044; *p* < 0.001; degree of freedom = 4. Hosmer–Lemeshow coefficient = 2.933; *p* = 0.710; degree of freedom = 5. Area under the ROC curve (AUC) = 0.854; confidence interval: 95% = (0.786–0.922); *p* < 0.001.

## Data Availability

The original contributions presented in this study are included in the article. Further inquiries can be directed to the corresponding authors.

## Abbreviations

The following abbreviations are used in this manuscript:

MS	Multiple sclerosis
RRMS	Relapsing–remitting multiple sclerosis
SPMS	Secondary progressive multiple sclerosis
PPMS	Primary progressive multiple sclerosis
PRMS	Progressive relapsing multiple sclerosis
DMT	Disease-modifying therapy
HBV	Hepatitis B virus
TLC	Total lymphocyte count
DMF	Dimethyl fumarate
